# The Appropriate First-Line Chemotherapy Regimen for Incurable Pancreatic Cancer in Clinical Practice: A Consideration of Patients' Overall Survival and Quality of Life

**DOI:** 10.1089/pancan.2021.0005

**Published:** 2021-08-06

**Authors:** Yasuko Murakawa, Kazunori Ootsuka, Makoto Abue

**Affiliations:** ^1^ Department of Cancer Chemotherapy and Miyagi Cancer Center, Natori, Japan.; ^2^ Department of Gastroenterology, Miyagi Cancer Center, Natori, Japan.

**Keywords:** first-line chemotherapy, incurable pancreatic cancer, overall survival, quality of life, retrospective study

## Abstract

**Purpose:** For incurable pancreatic cancer, the therapeutic goal is to prolong survival and maintain the quality of life (QOL). Unexpected outpatient consultation (OCT) and emergency hospitalization lead to QOL deterioration. The National Comprehensive Cancer Network (NCCN) guidelines recommend 5-fluorouracil/leucovorin, oxaliplatin, and irinotecan (FOLFIRINOX) and gemcitabine plus albumin-bound paclitaxel (nabPTX+GEM) as the preferred first-line regimens. Japanese clinical practice guidelines further recommend GEM and tegafur/gimeracil/oteracil potassium (S-1). Currently, no treatment strategy considers QOL at any stage during a patient's clinical course.

**Methods:** In this study, hospital-free survival (HFS), defined as the period without hospitalization and OCT, was introduced as a new indicator of the qualitative aspect of overall survival (OS). We compared OS, length of hospitalization (LOH), OCT, and HFS for the four first-line chemotherapy groups.

**Results:** No significant difference was observed in the median OS and HFS, nor was there a strong correlation between OS and LOH, based on the four first-line chemotherapy groups. In contrast, there were strong correlations between OS and OCT and between OS and HFS in all first-line chemotherapy groups. The ratio of OCT to OS was similar for mFOLFIRINOX and nabPTX+GEM. S-1 had the lowest OCT-to-OS ratio. The ratio of HFS to OS declined from highest to lowest in the order S-1, nabPTX+GEM, mFOLFIRINOX, and GEM.

**Conclusion:** Our findings suggested existence of correlation differences between OS and HFS between first-line mFOLFIRINOX and first-line nabPTX+GEM. In addition, a good HFS was obtained with S-1 alone in some cases. In the future, clinical trials for chemotherapy should examine QOL during the entire clinical course.

## Introduction

Pancreatic cancer is a lethal malignancy with poor prognosis.^1^ For unknown reasons, the incidence of pancreatic cancer increases with age. Recent data ranked the United States as third and Japan as fourth for estimated deaths due to pancreatic cancer among all cancer-related deaths.^2,3^ At the time of diagnosis, 80–85% of patients presented with advanced unresectable or distant metastatic disease.^4^ Furthermore, the response to conventional chemotherapy remained poor, and immune checkpoint inhibitors were ineffective for pancreatic cancer.^5^ The overall 5-year survival rate for patients with pancreatic cancer remains unchanged (10%), whereas the rate for colon cancer patients was >60% in 2020.^2^

In the National Comprehensive Cancer Network (NCCN) guidelines, version 2.2021, the combination of bolus and continuous 5-fluorouracil/leucovorin, oxaliplatin, and irinotecan (FOLFIRINOX); the combination of continuous 5-fluorouracil/leucovorin, oxaliplatin, and irinotecan (mFOLFIRINOX); and gemcitabine (GEM) plus albumin-bound paclitaxel (nabPTX+GEM) are preferred first-line regimens for locally advanced and metastatic pancreatic cancer. FOLFIRINOX and mFOLFIRINOX are recommended for patients with the Eastern Cooperative Oncology Group (ECOG) performance status (PS) scores 0–1, and nabPTX+GEM for patients with ECOG PS 0–2. In this guideline, other recommended regimens include GEM and capecitabine; second-line regimens are treatments that are not used as first-line regimens.^6^

In Japan, clinical practice guidelines for pancreatic cancer recommend FOLFIRINOX, nabPTX+GEM, GEM, and tegafur/gimeracil/oteracil potassium (S-1) with similar efficacy as first-line chemotherapy for patients with incurable pancreatic cancer.^7^ Treatment with mFOLFIRINOX has proven safe with similar effects to FOLFIRINOX.^8^

The standard chemotherapy presented in these guidelines is derived from the result of several clinical trials on the safety and efficacy of chemotherapy. Generally, safety is evaluated by the frequency and intensity of the adverse effects of the treatments. Effectiveness is evaluated by overall survival (OS) or progression-free survival (PFS).^9^

In incurable pancreatic cancer, the therapeutic goal of an oncologist is not to achieve cure but rather to control symptoms, prevent complications, prolong survival, and maintain as high a quality of life (QOL) as possible.^10^ QOL is most commonly evaluated using a questionnaire. However, it is difficult to administer questionnaires intermittently, particularly in patients with a poor general condition. The chemotherapy for cancer patients might lead to frequent hospitalization and outpatient consultation (OCT) due to the treatment itself or its adverse effects, thereby decreasing QOL.^11^

At present, no treatment strategy considers QOL during a patient's entire clinical course. The aim of this study was to determine the appropriate treatment strategy by analyzing a series of chemotherapy choices, treatment periods, OS, total length of hospitalization (LOH), and total OCT. Hospital-free survival (HFS), defined as the period without hospitalization and OCT, was introduced as a new indicator of the qualitative aspect of OS and was compared with OS.^12^

## Materials and Methods

In this study, we retrospectively evaluated 65 patients with pancreatic cancer who attended the Miyagi Cancer Center (Natori, Japan) between April 2014 and December 2018.

All patients were histologically confirmed, with PS 0–2, and diagnosed as incurable pancreatic ductal adenocarcinoma cancer due to distant metastasis or locally advanced tumor by computed tomography. Patients underwent chemotherapy for at least 30 days. We collected data from electric medical records between April 1, 2014 and December 31, 2020. The analysis data were recommended prognostic variables for first-line chemotherapy^13^ (e.g., age, gender, T-factor, N-factor, ascites, liver metastasis, and lung metastasis), chemotherapy regimens, treatment period, OS, and the variables related to QOL (e.g., LOH and OCT).

The ethics committee of the Miyagi Cancer Center approved this study (Approval No. 4). All procedures were performed according to the ethical standards of the institutional and national research committees and the 1964 Helsinki Declaration and its later amendments or comparable ethical standards.

According to the local ethics policy for the retrospective analysis of our own anonymized clinical data, informed consent, with an opt-out option, was obtained from all patients.

### Statistical analysis

In this study, we utilized the chi-square test to compare the clinicopathological characteristics (e.g., age, gender, tumor site, T-factor, N-factor, ascites, liver metastasis, and lung metastasis) among the first-line chemotherapy regimens. Kaplan–Meier analysis and log-rank tests were used for the survival comparisons. Multivariate Cox regression analysis was performed to adjust for confounding factors of survival outcomes. A two-tailed *p* <0.05 was considered significant. The correlation between OS and LOH, OS and OCT, and OS and HFS were examined using scatterplots; a coefficient of determination, *r*^2^ ≥ 0.5 was considered a strong correlation, whereas 0.5 > *r*^2^ ≥ 0.1 was considered a moderate correlation.

All statistical analyses were performed using the IBM Statistical Package for the Social Sciences Statistics for Windows, version 24 (IBM Corp., Armonk, NY).

## Results

By the end of December 2020, of the 65 incurable pancreatic cancer patients, 59 had died. Of the seven surviving patients, four had only palliative treatment, and three continued chemotherapy.

FOLFIRINOX was not recommended as a first-line chemotherapy for patients >70 years of age due to the complexity of continuous administration for 48 h and the possibility of strong adverse effects. Hence, patients with FOLFININOX as the first-line chemotherapy were younger (≥70 years/<70 years: 0/16), whereas most of those on S-1 were elderly (≥70 years/<70 years: 15/5); patients on nabPTX+GEM were divided almost evenly between the two groups (≥70 years/<70 years: 11/12).

There was no significant difference in first-line chemotherapy by gender, T-factor, N-factor, ascites, liver metastasis, and lung metastasis (Table 1).

**Table 1.** The Characteristics of Patients with Incurable Pancreatic Cancer by First-Line Chemotherapy Regimens

First regimen	mFOLFIRINOX	nabPTX+GEM	S-1	GEM	p
(N)	(16)	(23)	(20)	(6)	
Variable					
Age: ≥70/<70	0/16	11/12	15/5	3/3	<0.001^*^
Gender: F/M	7/9	9/14	8/12	2/4	0.979
T-factor: 2/3/4	3/5/8	3/6/14	4/8/8	1/0/5	0.553
N-factor: −/+	7/9	9/14	15/5	4/5	0.084
Ascites: −/+	14/2	15/8	18/2	4/2	0.156
Liver metastasis: −/+	9/7	15/8	12/8	6/0	0.265
Lung metastasis: −/+	11/5	21/2	17/3	5/1	0.321

^*^ *p* < 0.05.

F, female; M, male; mFOLFIRINOX, 5-fluorouracil/leucovorin, oxaliplatin, and irinotecan; nabPTX+GEM, gemcitabine plus albumin-bound paclitaxel; S-1, tegafur/gimeracil/oteracil potassium.

When the first-line chemotherapy was mFOLFIRINOX, 50% (8/16) of patients had nabPTX+GEM as the second-line chemotherapy, whereas 44% (7/16) had no second-line chemotherapy. When the first-line chemotherapy was nabPTX+GEM, 26% (6/23) of patients had mFOLFIRINOX as the second-line chemotherapy, whereas 52% (12/23) had no second-line chemotherapy. When the first-line chemotherapy was S-1, a second-line chemotherapy was not administered in 90% (18/20) of the patients (Table 2).

**Table 2.** First-Line and Second-Line Regimen in Patients with Incurable Pancreatic Cancer

First regimen	mFOLFIRINOX	nabPTX+GEM	S-1	GEM
Second regimen				
mFOLFIRINOX		6 (26.1%)	0	0
nabPTX+GEM	8 (50.0%)		0	0
*S*-1	0	0		2 (33.3%)
GEM	1 (6.3%)	3 (13.0%)	0	
GEM+S-1	0	2 (8.7%)	2 (10.0%)	1 (16.7%)
FOLFIRI	0	0	0	1 (16.7%)
(−)	7 (43.8%)	12 (52.2%)	18 (90.0%)	2 (33.3%)
Total	16 (100%)	23 (100%)	20 (100%)	6 (100%)

**FIG. 1.**
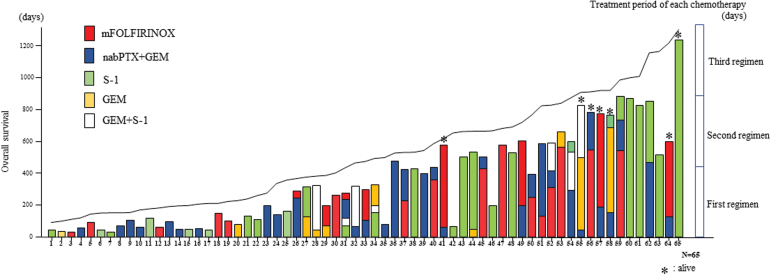
OS increased as the chemotherapy period increased. Thirty-nine patients received only first-line chemotherapy, whereas 26 patients further received second-line chemotherapy. Of the 26 patients, the longest treatment period occurred during the first line, second line, and third line in 11, 13, and 2 patients, respectively. Some patients who received only S-1 showed long-term survival. OS, overall survival; S-1, tegafur/gimeracil/oteracil potassium.

As shown in Figure 1, OS increased as the chemotherapy period increased. In other words, patients in physically good condition who could continue chemotherapy achieved a longer OS. Thirty-nine patients received only first-line chemotherapy, whereas 26 patients further received second-line chemotherapy. Of these 26 patients, the longest treatment period occurred during the first-line, second-line, and third-line chemotherapy in 11, 13, and 2 patients, respectively. Some patients who received only S-1 showed a long-term survival of 3 years or more.

In Kaplan–Meier analysis, no significant difference was found between the first-line chemotherapy regimens in median OS (mFOLFIRINOX, 19.0 months vs. nabPTX+GEM, 15.3 months; *p* = 0.689, mFOLFIRINOX, 19.0 months vs. S-1, 14.0 months; *p* = 0.978, and mFOLFIRINOX, 19.0 months vs. GEM, 12.0 months; *p* = 0.051) and median HFS (mFOLFIRINOX, 15.8 months vs. nabPTX+GEM, 10.0 months; *p* = 0.655, mFOLFIRINOX, 15.8 months vs. S-1, 10.5 months; *p* = 0.675, and mFOLFIRINOX, 15.8 months vs. GEM, 10.4 months; *p* = 0.064) (Supplementary Figs. S1 and S2).

First-line mFOLFIRINOX was significantly and independently associated with a longer OS, compared with first-line GEM (hazard ratio [HR]: 4.36, 95% confidence interval [95% CI]: 1.40–13.58, *p* < 0.05). First-line mFOLFIRINOX might not be significantly associated with a longer OS, compared with first-line nabPTX+GEM and S-1. N-factor (+) was significantly associated with a shorter OS, compared with the N-factor (−) (HR: 0.43, 95% CI: 0.22–0.84, *p* < 0.05). Liver metastasis (+) was significantly associated with a shorter OS, compared with liver metastasis (−) (HR: 0.21, 95% CI: 0.09–0.46, *p* < 0.005) (Table 3).

**Table 3.** Multivariate Cox Regression Analysis of Overall Survival

Variable	HR (95.0% CI)	p-Value
First-line chemotherapy		
nabPTX+GEM vs. mFOLFIRINOX	0.98 (0.42–2.27)	0.97
S-1 vs. mFOLFIRINOX	2.37 (0.91–6.20)	0.77
GEM vs. mFOLFIRINOX	4.36 (1.40–13.58)	<0.05^*^
Age (<70 vs. ≥70) years	1.46 (0.67–3.15)	0.34
Gender (female vs. male)	0.98 (0.52–1.84)	0.94
Tumor site (head vs. body/tail)	1.86 (0.86–3.80)	0.12
T-factor		
T2 vs. T4	0.47 (0.20–1.10)	0.08
T3 vs. T4	0.42 (0.17–1.03)	0.06
N-factor (+/−)	0.43 (0.22–0.84)	<0.05^*^
Ascites (+/−)	0.50 (0.23–1.09)	0.08
Liver metastasis (+/−)	0.21 (0.09–0.46)	<0.005^*^
Lung metastasis (+/−)	0.55 (0.25–1.23)	0.15

^*^ *p* < 0.05.

CI, confidence interval; HR, hazards ratio.

First-line mFOLFIRINOX was significantly associated with a longer HFS, compared with first-line GEM (HR: 3.45, 95% CI: 1.23–10.57, *p* < 0.05). First-line mFOLFIRINOX was nonsignificantly associated with a longer HFS, compared with first-line nabPTX+GEM and S-1. Body/tail tumor was significantly associated with a longer HFS, compared with head tumor (HR: 2.11, 95% CI: 1.03–4.32, *p* < 0.05). T2 and T3 (T-factors) were significantly associated with a longer HFS, compared with T4 (T2 [HR: 0.41, 95% CI: 0.17–0.99, *p* < 0.05], T3 [HR: 0.32, 95% CI: 0.13–0.82, *p* < 0.05]). N-factor (+) was significantly associated with a shorter HFS, compared with the N-factor (−) (HR: 0.40, 95% CI: 0.20–0.78, *p* < 0.01). Liver metastasis (+) was significantly associated with a shorter HFS, compared with liver metastasis (−) (HR: 0.19, 95% CI: 0.09–0.44, *p* < 0.001) (Table 4).

**Table 4.** Multivariate Cox Regression Analysis of Hospital-Free Survival

Variable	HR (95.0% CI)	p-Value
First-line chemotherapy		
nabPTX+GEM vs. mFOLFIRINOX	0.91(0.40–2.07)	0.82
S-1 vs. mFOLFIRINOX	1.92 (0.75–4.94)	0.18
GEM vs. mFOLFIRINOX	3.45 (1.23–10.57)	<0.05^*^
Age (<70 vs. ≥70) years	1.39 (0.64–3.03)	0.41
Gender (female vs. male)	1.02 (0.54–1.93)	0.95
Tumor site (head vs. body/tail)	2.11 (1.03–4.32)	<0.05^*^
T-factor		
T2 vs. T4	0.41 (0.17–0.99)	<0.05^*^
T3 vs. T4	0.32 (0.13–0.82)	<0.05^*^
N-factor (+/−)	0.40 (0.20–0.78)	<0.01^*^
Ascites (+/−)	0.61 (0.28–1.31)	0.20
Liver metastasis (+/−)	0.19 (0.09–0.44)	<0.001^*^
Lung metastasis (+/−)	0.59 (0.26–1.31)	0.19

^*^ *p* < 0.05.

**FIG. 2.**
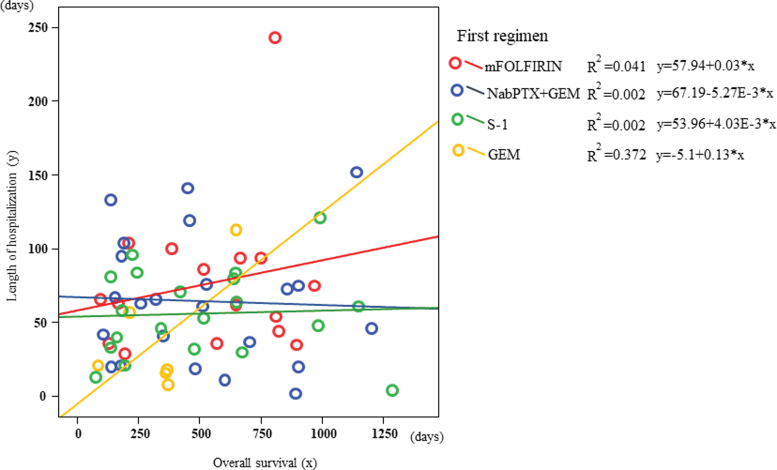
In the correlation between OS (*x*-axis) and LOH (*y*-axis) among first-line chemotherapy regimens, a moderate correlation was observed in GEM; however, no correlation was observed between mFOLFIRINOX, nabPTX+GEM, and S-1. LOH, length of hospitalization; mFOLFIRINOX, 5-fluorouracil/leucovorin, oxaliplatin, and irinotecan; nabPTX+GEM, gemcitabine plus albumin-bound paclitaxel.

The correlation between OS and LOH among first-line chemotherapy regimens was examined using scatterplot analysis. In GEM, a moderate correlation was observed between OS (*x*-axis) and LOH (*y*-axis) (coefficient of determination: *r*^2^ = 0.372, *y* = −5.1 + 0.13*x*); however, no correlation was observed between mFOLFIRINOX, nabPTX+GEM, and S-1 (*r*^2^: 0.041, 0.002, 0.002, respectively) (Fig. 2).

**FIG. 3.**
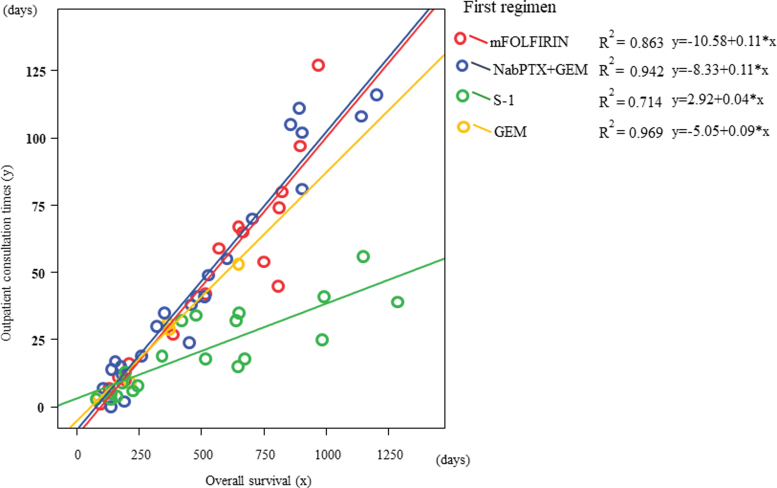
Strong correlations occurred between OS (*x*-axis) and OCT (*y*-axis) in all first-line chemotherapy groups. The ratio of OCT to OS, mFOLFIRINOX, and nabPTX+GEM, respectively, were the same, followed by that of GEM and S-1; from the highest to the lowest, in that order. OCT, outpatient consultation.

Strong correlations were observed between OS (*x*-axis) and OCT (*y*-axis) in all first-line chemotherapy groups. The ratios of OCT to OS for mFOLFIRINOX and nabPTX+GEM were the same, followed by that of GEM and S-1; from the highest to the lowest (mFOLFIRINOX: *r*^2^ = 0.863, *y* = −10.58 + 0.11*x*; nab-PTX+GEM: *r*^2^ = 0.942, *y* = −8.33 + 0.11*x*; S-1: *r*^2^ = 0.714, *y* = 2.92 + 0.04*x*; GEM: *r*^2^ = 0.969, *y* = −5.05 + 0.09*x*) (Fig. 3).

**FIG. 4.**
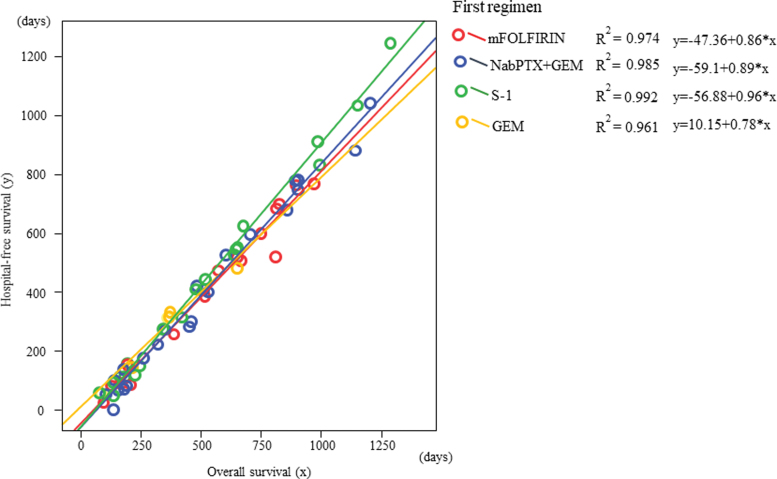
Strong correlations occurred between OS (*x*-axis) and HFS (*y*-axis) in all first-line chemotherapy groups. The ratio of HFS to OS, S-1, nabPTX+GEM, mFOLFIRINOX, and GEM, from the highest to the lowest, was reported. HFS, hospital-free survival.

Strong correlations were observed between OS (*x*-axis) and HFS (*y*-axis) in all first-line chemotherapy groups. The ratios of HFS to OS, S-1, nabPTX+GEM, mFOLFIRINOX, and GEM, from the highest to the lowest, were reported (mFOLFIRINOX: *r*^2^ = 0.974, *y* = −47.36 + 0.86*x*; nabPTX+GEM: *r*^2^ = 0.985, *y* = −59.1 + 0.89*x*; S-1: *r*^2^ = 0.992, *y* = −56.88 + 0.96*x*; GEM: *r*^2^ = 0.961, *y* = 10.15 + 0.78*x*) (Fig. 4).

## Discussion

When evaluating first-line chemotherapy for incurable pancreatic cancer, it was considered effective to analyze both HFS and OS.

Many mandatory prognostic factors are recommended for unresectable pancreatic cancer,^13^ among which liver metastasis has been reported as the most important.^14,15^ Moreover, lymph node metastasis may predict the likelihood of survival.^16^ In this study, patients with liver and lymph node metastases had a shorter OS and HFS compared with those without these metastases. A shorter HFS occurred more frequently in patients with T-factor, T4, and head tumor. The aforementioned result may be explained by the fact that patients with pancreatic head tumor that extends to the celiac or mesenteric artery tend to develop obstructive jaundice that requires frequent OCT or hospitalization.

In many clinical studies, FOLFIRINOX and nabPTX+GEM as first-line chemotherapy resulted in longer OS than GEM alone.^17–19^ Even in clinical practice, several reports suggest that OS is prolonged by introducing FOLFIRINOX and nabPTX+GEM.^20,21^ At present, no conclusion has been reached regarding which treatment is better.^22^

In second-line chemotherapy, FOLFIRINOX requires careful observation for treatment-related hematological toxicities after GEM-including regimens.^23^ It has also been reported that liposomal irinotecan +5-FU/LV regimen could be used to prolong OS and maintain QOL as a second-line chemotherapy in patients with GEM refractory.^24,25^ The importance of developing further strategies for individualized second-line treatment regimens based on the first-line chemotherapy has been noted.^26^

QOL studies in patients during chemotherapy are often conducted using questionnaires, such as the European Organization for the Research and Treatment of Cancer Quality of Life Questionnaire Core 30 (EORCT QLQ-30) and Functional Assessment of Cancer Therapy-General, during a specific treatment period.^27–29^ At present, it is difficult to examine the trajectory of QOL over the entire course of a patient, from the start of chemotherapy till death.

Cancer chemotherapy utilization has shifted from the hospital to an outpatient setting. Outpatient chemotherapy has the advantage of providing an opportunity to respect a patient's wish to avoid hospitalization and enhance a patient's physical comfort and psychological well-being, thereby promoting a good QOL.^30^ In contrast, outpatient waiting time is cited as a factor that lowers a patient's level of satisfaction with treatment regimens. Besides, unexpected outpatient visits and emergency hospitalization (occasionally) due to cancer progression and adverse effect of chemotherapy lead to a deteriorating QOL.^31^ Patients with pancreatic cancer are reported to have frequent unexpected hospitalizations.^32^

In this study, the main purposes of the outpatient visits were to (1) receive palliative chemotherapy, (2) undergo treatment evaluation, such as computed tomography or magnetic resonance imaging, and (3) manage the exacerbation of cancer and the adverse effects of chemotherapy. The main purposes of hospitalization were to (1) receive the first chemotherapy regimen, if needed, (2) manage the problems related to bile duct stents for improvement of obstructive jaundice, and (3) control symptoms that are difficult to manage on OCT.

The treatment schedule of mFOLFIRINOX was every 2 weeks; however, in clinical practice, the treatment interval was often every 3 weeks due to the adverse effects of these anticancer agents. The treatment schedule of nabPTX+GEM and GEM was every 4 weeks, with an anticancer agent administered on days 1, 8, and 15. However, it was frequently administered every 2 weeks due to the adverse effects of these anticancer agents. The treatment schedule of S-1 was every 3 weeks, consisting of 2 weeks of oral administration and the following week off.

In this study, there was no strong correlation between OS and LOH, depending on the first-line chemotherapy group. However, the LOH may reflect the diversity of individual pancreatic cancer progression.

There was a strong correlation between OS and OCT in all types of first-line chemotherapy groups. mFOLFIRINOX and nabPTX+GEM had a similar ratio of OCT to OS; however, similar ratios were derived from both the first-line and subsequent chemotherapy. S-1 had the lowest ratio of OCT to OS because second-line chemotherapy was infrequent, and outpatient visits occurred every 3 weeks. There was a strong correlation between OS and HFS in all first-line chemotherapy groups, and the ratio of HFS to OS declined from highest to lowest in the order S-1, nabPTX+GEM, mFOLFIRINOX, and GEM.

This study has several limitations. We conducted a retrospective study with only 65 cases from a single facility. The chemotherapy had various modifications, such as reduced drug dosage and extended treatment interval, unlike clinical trials. This study included short survival cases with a treatment period of ∼40 days. In addition, there is an age bias in treatment groups. HFS is not sufficient to evaluate QOL during the patients' entire clinical course because it is not a QOL based on the patient's own evaluation.

## Conclusion

The study findings suggested existence of correlation differences between OS and HFS between first-line mFOLFIRINOX and first-line nabPTX+GEM. Moreover, a good HFS was obtained with S-1 alone in some cases.

In the future, when conducting a randomized controlled trial on first-line chemotherapy regimens of pancreatic cancer, it may be necessary to examine HFS.

## Supplementary Material

Supplemental data

Supplemental data

## Declarations

### Availability of data and materials

The raw data of this study are shared in Fig share (https://doi.org/10.6084/m9.figshare.14472753).

## Abbreviations Used

ECOG: Eastern Cooperative Oncology Group
HFS: hospital-free survival
LOH: length of hospitalization
mFOLFIRINOX: 5-fluorouracil/leucovorin, oxaliplatin, and irinotecan
nabPTX+GEM: gemcitabine plus albumin-bound paclitaxel
NCCN: National Comprehensive Cancer Network
OCT: outpatient consultation
OS: overall survival
PFS: progression-free survival
PS: performance status
QOL: quality of life
S-1: tegafur/gimeracil/oteracil potassium
