# Supplementary figures and images for: The Appropriate First-Line Chemotherapy Regimen for Incurable Pancreatic Cancer in Clinical Practice: A Consideration of Patients' Overall Survival and Quality of Life
Source: J Pancreat Cancer. 2021 Aug 6;7(1):48–56. doi: 10.1089/pancan.2021.0005 (PMC8655810; doi:10.1089/pancan.2021.0005)

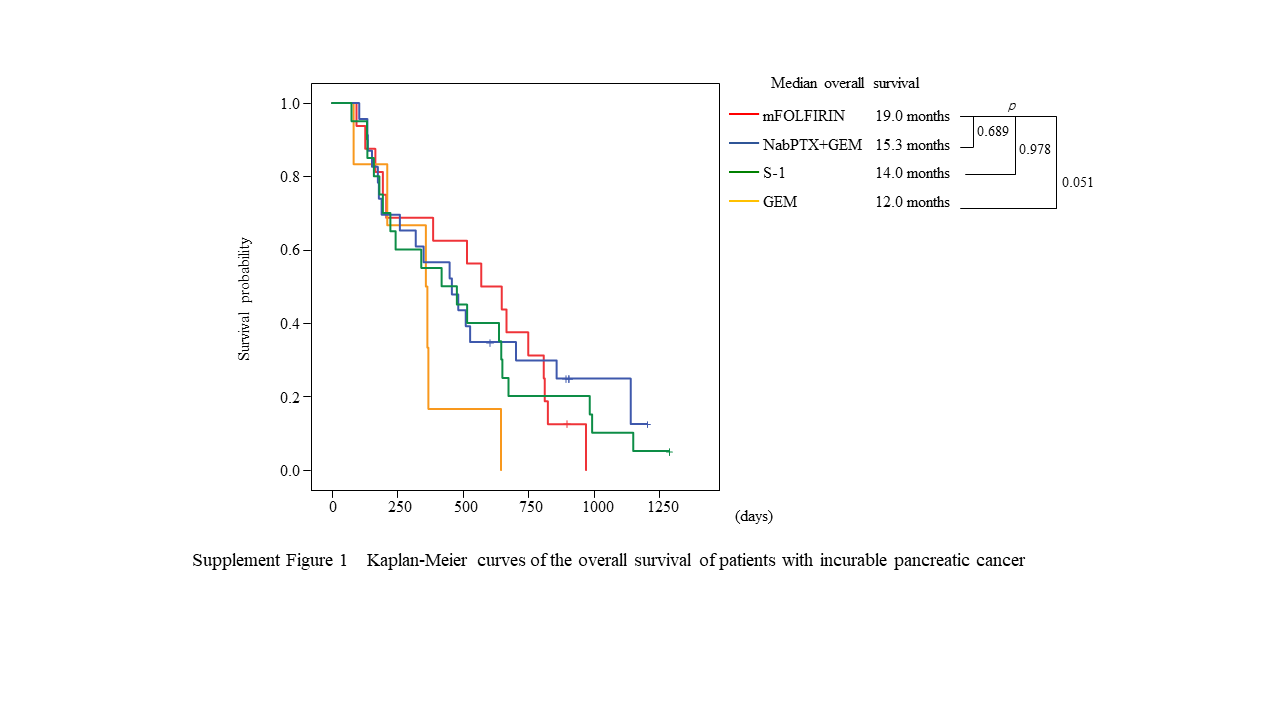

Supplement: Supplemental data [file Supp_Fig1.TIF]

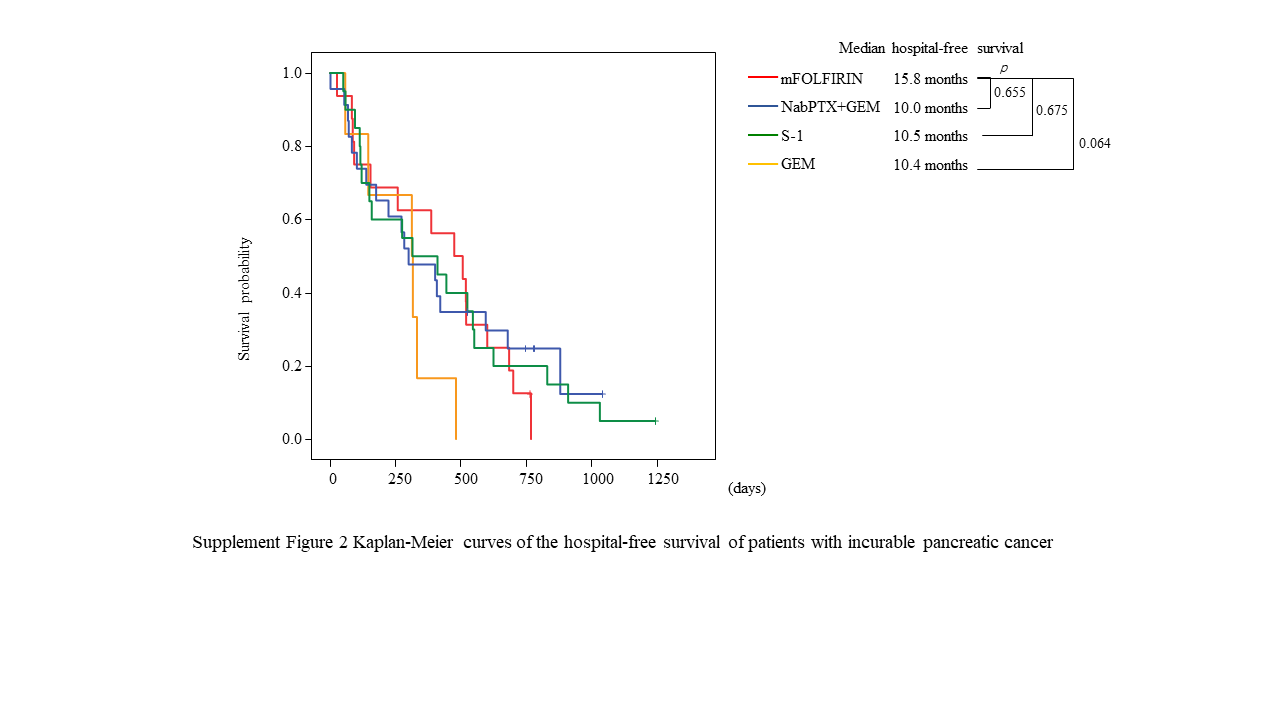

Supplement: Supplemental data [file Supp_Fig2.TIF]
